# Amyloid Beta Biomarkers and Their Association with Cognitive Impairment Across Vitamin D Strata: A Cross-Sectional Study in an Older Adult South Indian Population

**DOI:** 10.3390/nu18182942

**Published:** 2026-09-08

**Authors:** Rajalakshmi Ramashetty, Rimshia Naaz, Ramya C M, SubbaRao V. Madhunapantula, Chinnappa A. Uthaiah

**Affiliations:** 1 Department of Physiology, Jagadguru Sri Shivarathreeshwara (JSS) Medical College, JSS Academy of Higher Education & Research (JSS AHER), Mysuru 570015, Karnataka, India; 2 Center of Excellence in Molecular Biology and Regenerative Medicine (A DST-FIST Supported Center), Department of Biochemistry (A DST-FIST Supported Department), JSS Medical College, JSS Academy of Higher Education & Research (JSS AHER), Mysuru 570015, Karnataka, India; mvsstsubbarao@jssuni.edu.in (S.V.M.);

**Keywords:** vitamin D, cognition, aging, amyloid beta, oxidative stress

## Abstract

**Introduction:** Vitamin D status has been investigated as a modifiable risk factor with cognitive decline. However, studies pertaining to this remain inconsistent across populations, and there are fewer studies associating vitamin D status, cognitive decline, amyloid markers and oxidative stress markers in older adults. Therefore, this study was conducted to analyze the association between these parameters. **Methodology:** This cross-sectional study included 145 older adults aged between 51 and 80 years. Participants were categorized based on serum vitamin D levels into sufficient (n = 53), insufficient (n = 61), and deficient (n = 31) groups. Cognitive performance was assessed using the Modified Mini Mental State (3MS) test. Plasma levels of Aβ 1-40 and Aβ 1-42 and oxidative stress markers (TBARS, GPx, SOD) were measured. Statistical analysis was performed using ANOVA and Spearman’s correlation. **Results:** Results showed that the vitamin D concentrations did not differ significantly between cognitively impaired and normal individuals within vitamin D strata (vitamin D sufficient, insufficient and deficient groups). Plasma Aβ 1-40 levels were significantly elevated in cognitively impaired participants across all vitamin D groups. A reduced Aβ 1-42/Aβ 1-40 ratio was observed in sufficient and insufficient Vitamin D groups. Additionally, GPx levels were significantly higher in the vitamin D-deficient group. Correlation analysis showed that 3MS scores were negatively associated with age (r = −0.2607, *p* = 0.0015), Aβ 1-40 (r = −0.4016, *p* = 0.0001), and Aβ 1-42 (r = −0.3025, *p* = 0.0002), and positively correlated with Aβ 1-42/Aβ 1-40 ratio (r = 0.2176, *p* = 0.0086). **Conclusions:** These findings suggest that cognitive impairment in older adults is strongly associated with amyloid burden, oxidative stress, and age, whereas vitamin D status was not directly correlated with cognitive performance in this cohort.

## 1. Introduction

Vitamin D, a fat-soluble secosteroid hormone essentially known for its action in bone metabolism and calcium homeostasis, has now been increasingly recognized in brain health [1,2]. This is due to the presence of vitamin D receptors (VDR) distributed widely in brain regions responsible for cognition like the hippocampus and limbic system [3,4]. Activation of VDR signaling is stated to regulate neuronal differentiation, synaptic plasticity and expression of neurotrophic factors which are critical for learning and memory [5,6].

Accumulating epidemiological evidence indicates that vitamin D deficiency may be associated with cognitive decline and dementia [7,8,9]. Consistent with this, many prospective cohort studies have shown that individuals with lower serum levels of 25-hydroxyvitamin D [25(OH)D] are at increased risk of cognitive decline over follow-up periods spanning up to several years [10,11]. Vitamin D deficiency has been linked with reduced memory consolidation, cognitive processing deceleration and weakened executive functioning [12]. Observational studies consistently showed a strong association between low 25(OH)D and increased susceptibility for cognitive decline and Alzheimer’s disease (AD) [13]. Randomized controlled trials (RCTs) have shown mixed responses; some studies have shown improvements in cognitive outcomes following vitamin D intervention, especially in deficient populations [14,15] and other studies have shown no association [16,17,18]. The biological mechanisms linking vitamin D deficiency to cognitive decline are multifactorial, among which the amyloid pathway involving the isoforms Aβ 1-40 and Aβ 1-42 is crucial. Studies have shown that the Aβ 1-42/Aβ 1-40 ratio serves as a key biomarker of Alzheimer’s disease pathology, with lower ratios indicating increased cerebral deposition of Aβ 1-42 [19,20]. Apart from this, vitamin D deficiency has also been linked to impaired amyloid clearance and increased cerebral accumulation [21]. Preclinical models suggest that vitamin D activation may regulate neuronal differentiation and synaptic plasticity [22]. Furthermore, experimental evidence indicates that vitamin D may facilitate amyloid clearance through the blood–brain barrier [23,24]. While our current cross-sectional study cannot demonstrate these causal mechanisms directly, it aims to explore the associations between vitamin D status, circulating amyloid markers and cognitive performance in older adults of South India to see if these clinical patterns align with hypothesized biological pathways.

Oxidative stress (OS) constitutes an additional critical contributor to neurodegenerative processes and cognitive decline [25]. Excessive production of reactive oxygen species (ROS) can harm neuronal membranes, proteins and nucleic acids resulting in synaptic dysfunction, neuronal loss, Aβ aggregation and cognitive deterioration [26]. Vitamin D expresses antioxidant properties by downregulating ROS production and enhancing the antioxidant enzyme activity of superoxide dismutase (SOD) and glutathione peroxidase (GPx) [27,28]. Vitamin D deficiency may aggravate oxidative stress-induced injury, promote amyloid deposition, and amplify neuroinflammatory cascades, augmenting neurodegeneration [29]. Animal studies reveal that vitamin D intervention mitigates oxidative stress, reduces Aβ load, and ameliorates cognitive performance in AD models [30,31].

Despite the mounting global literature on these pathways, several critical research gaps remain unaddressed. First, studies exploring the clinical and biochemical relationships between vitamin D status, amyloid burden, and oxidative stress are exceedingly sparse in Indian populations, despite a rapid, alarming rise in the prevalence of hypovitaminosis D among older adults in India. Second, there is a distinct lack of clinical studies that simultaneously evaluate cognitive performance alongside a comprehensive, parallel panel of both circulating amyloid-beta markers (Aβ 1-40, Aβ 1-42, and their ratio) and key antioxidant enzymes (SOD, GPx, and TBARS) in a single well-defined cohort. Finally, older cohorts in developing regions often carry a substantial, unaddressed background of metabolic dysregulation characterized by chronic hyperglycemia and insulin resistance, which can independently saturate shared amyloid-degrading enzymes and promote oxidative injury, representing an unmeasured confounding background that must be evaluated.

To address these critical gaps, the present cross-sectional study was designed to investigate the association between serum 25(OH)D levels, global cognitive performance, circulating amyloid-beta biomarkers and systemic oxidative stress markers in an older South Indian population By characterizing the relationships between these clinical parameters, our study aims to clarify the clinical and biochemical relevance of vitamin D status in the context of late-life cognitive health and heavy metabolic burden, paving the way for more targeted multidimensional therapeutic strategies.

## 2. Methodology

**Study design:** This cross-sectional study utilized a dual-recruitment strategy to enroll participants. Subjects were recruited from both the outpatient departments of a tertiary care hospital in Mysuru and from the surrounding suburban community through local outreach post-approval from the Institutional Ethics Committee (JSSMC/IEC/14/3712/2016-17). Out of 200 subjects screened, 145 individuals aged between 51 and 80 years were enrolled according to clearly defined inclusion and exclusion criteria. The exclusion process eliminated individuals who had diabetes-related complications, coronary artery disease, endocrine disorders, a history of stroke, psychiatric disorders, carcinomas and those receiving vitamin D supplementation. Consent was acquired and documented from participants, ensuring ethical adherence. Relevant clinical history was collected for all participants, alongside a general physical examination. Anthropometric measurements including height, weight, waist circumference and hip circumference were recorded following standardized procedures. Blood pressure was also measured.

**Blood Sample Collection:** The patient’s blood sample was collected by a trained phlebotomist using the venepuncture method. Approximately 6.0 mL of blood in the fasting state was collected in plain BD Vacutainers (Roche Diagnostics Corporation, Indianapolis, IN, USA) (Cat no: 367820), from which serum was subsequently separated and aliquoted for further biochemical analysis.

**Glycemic Parameters:** Fasting blood sugar (FBS) was measured using a hexokinase-based method with the Gluc3 kit (Cat no: 04404483-190) on a Cobas c 501 automated analyzer (Roche Diagnostics, Indianapolis, IN, USA). Fasting serum insulin levels were measured using the Elecsys Insulin kit (Cat no: 12017547-122) on the Cobas e 601 analyzer. HbA1c was measured using the Biorad D-10 Hemoglobin Testing System (Cat no: 220-0101; Bio-Rad, Hercules, CA, USA). HOMA-IR (homeostatic model assessment of insulin resistance) was calculated using the formula: HOMA-IR = fasting insulin (µIU/mL) × FBS (mg/dL)/405.

**Lipid Profile:** The lipid parameters were analyzed using the Cobas c 501 autoanalyzer (Roche Diagnostics, USA) with the following kits: triglycerides (Cat no: 20767107-322), HDL-C (Cat no: 04399803-190), LDL-C (Cat no: 07005717-190) and total cholesterol (Cat no: 03039773-190).

**Amyloid Beta Quantification:** Human serum Aβ_1-40_ levels were measured using a commercially available competitive ELISA kit (Cat no: CEA864Hu; Cloud Clone, Katy, TX, USA), which has a reported detection range of 12.35–1000 pg/mL and a minimum sensitivity dose of <5.95 pg/mL. Human serum Aβ_1-42_ levels were measured using a commercially available high-sensitivity competitive ELISA kit (Cat no: HEA946Hu; Cloud Clone, USA), with a reported detection range of 3.70–300 pg/mL and a minimum detectable dose of <1.24 pg/mL. Both assays were performed as per the manufacturer’s protocols. According to the manufacturer, these kits exhibit high specificity for their respective targets with no significant cross-reactivity or interference observed between Aβ 1-40, Aβ 1-42, or other analogous peptides. To mitigate technical challenges such as the “hook effect” and to ensure high precision, all serum samples were assayed in duplicate.

**Vitamin D levels:** Serum 25(OH)D levels were quantified using a fully automated electrochemiluminescence immunoassay (ECLIA) system, Cobas e 601 (Roche Diagnostics, USA) with the Vitamin D Total Elecsys kit (Cat no: 05894913-190). Participants were stratified based on their serum vitamin D levels into specific categories: vitamin D deficient (<20 ng/mL), vitamin D insufficient (21–30 ng/mL) and vitamin D sufficient (>31 ng/mL).

**Oxidative Stress Markers:** SOD activity was assessed using a high-throughput SOD assay kit (Cat no: 7501-500-K; R&D Systems, Minneapolis, MN, USA). GPx activity was determined using a high-throughput GPx assay kit (Cat no: 7512-100-K; R&D Systems, USA) and TBARS was assessed using the MDA method described by Ghani et al. [32]. 

**Cognitive assessment:** All participants were subjected to the Modified Mini-Mental State Examination (3MS) [33]. Cognitive performance was assessed using the 100-point version of the 3MS. This version expands the traditional MMSE to improve sensitivity and provides a broader range of scores. The domains were weighted as follows: orientation (15 points)—includes temporal orientation (5 points) and spatial orientation (10 points); registration (3 points)—immediate recall of three words; attention and calculation (7 points)—assessed through mental reversal tasks (e.g., spelling a word backward); recall (9 points)—delayed recall of the three previously registered words, with points for spontaneous recall or recall with prompts; language and praxis (approximately 39 points)—includes naming, repetition, following a three-stage command, reading, writing and copying intersecting pentagons; and other components (remaining points)—includes tasks such as personal information, word fluency (naming animals) and similarities. Participants scoring below 80 were categorized as having cognitive impairment.

**Statistical Analysis**: Data compilation was performed using Microsoft Excel 2019, and subsequent analysis utilized JMP Pro Version 17 (JMP Statistical Discovery LLC, Cary, NC, USA). Descriptive statistics summarized the clinical, biochemical, and cognitive parameters. Continuous variables were depicted as mean with standard deviation (SD) and categorical variables as frequencies and percentages. The normality of continuous data was assessed via the Kolmogorov–Smirnov test. Group comparisons were performed using the following tests: for two-group comparisons, independent *t*-tests or Mann–Whitney U tests were employed; for comparisons among more than two groups, either one-way ANOVA or Kruskal–Wallis test were used. The chi-square test was applied to compare proportions of categorical variables. Spearman’s rank correlation test was used to check the associations between continuous nonparametric variables like vitamin D, cognitive scores, amyloid markers and oxidative stress markers. Logistic regression analysis was performed to show independent predictors of cognitive impairment. Odds ratios (ORs) with 95% confidence intervals (CIs) were reported and receiver operating characteristic (ROC) curve analysis assessed the diagnostic capacity of vitamin D and serum biomarkers in predicting cognitive impairment. A *p*-value of ≤0.05 was considered statistically significant.

## 3. Results

### 3.1. Participant Characteristics

**Figure 1:**
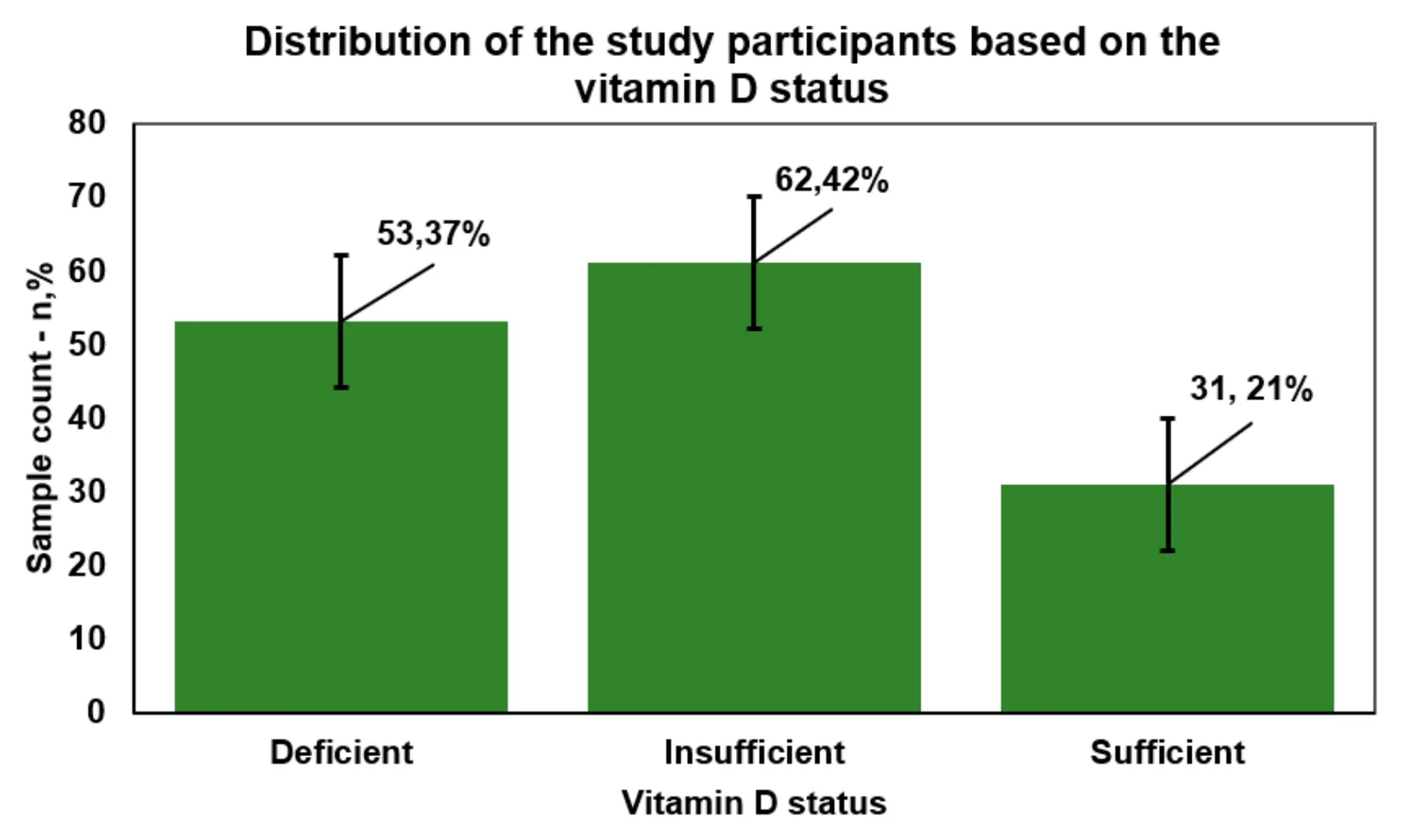
Depiction of the distribution of study participants based on vitamin D status.

A total of 145 subjects were enrolled in the study, comprising 94 females (64.83%) and 51 males (35.17%) (Table 1). The distribution of participants based on vitamin D status is presented in Figure 1. Among the participants, 31 (21.38%) were classified as vitamin D deficient (≤20 ng/mL), 61 (42.07%) as vitamin D insufficient (21–30 ng/mL) and 53 (36.55%) as vitamin D sufficient (≥ 31 ng/mL) indicating a high prevalence of low vitamin D levels in the study population, with Vitamin D insufficiency being the most prevalent and statistically significant (χ^2^ = 13.10, *p* = 0.0014).

**Figure 2:**
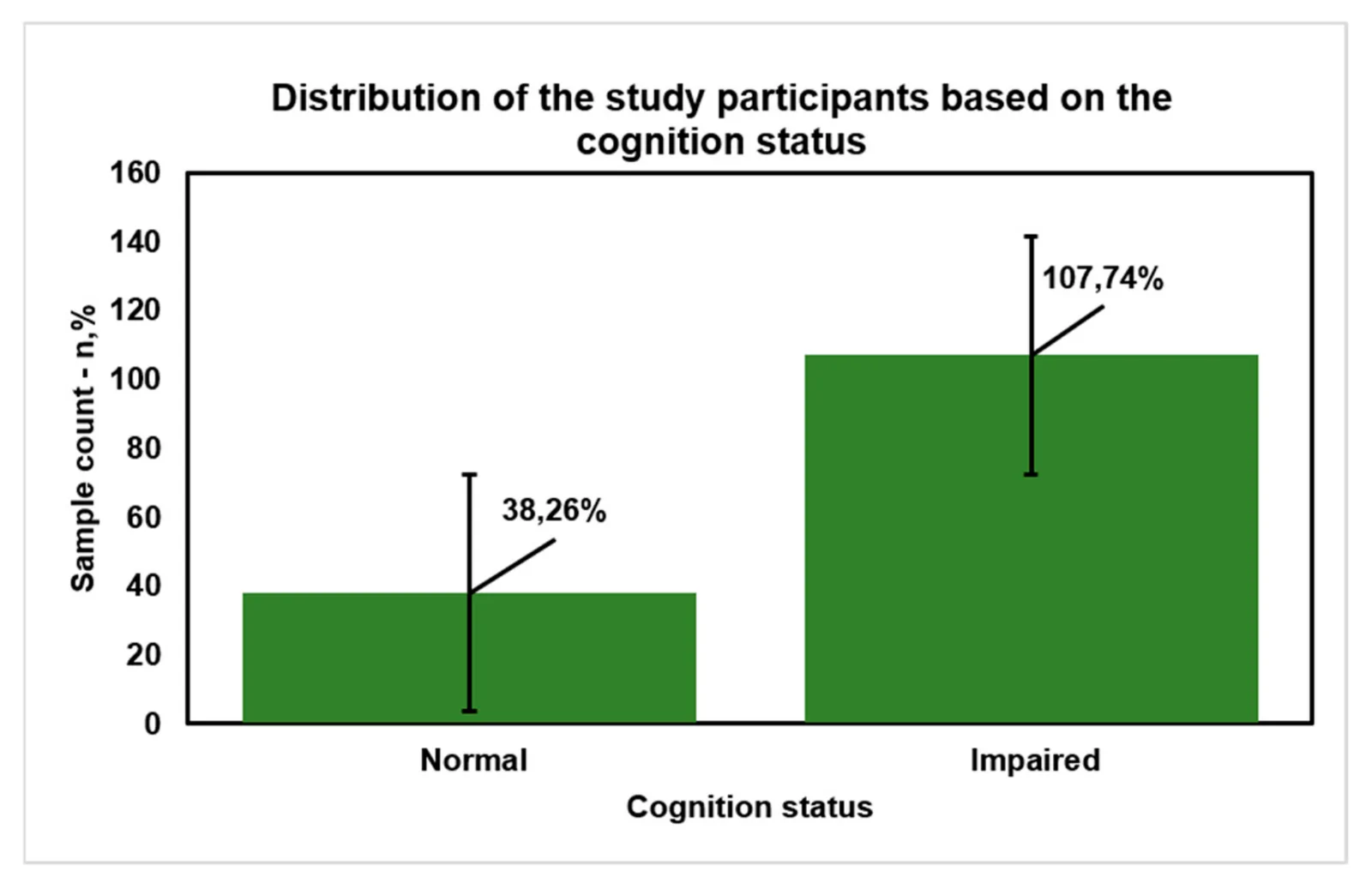
Depiction of the distribution of study participants based on cognition status.

In the study population (n = 145), cognitive impairment was highly prevalent. As presented in Figure 2, 74% of participants (n = 107) were classified as having cognitive impairment, while only 26% (n = 38) demonstrated normal cognitive function as per the 3MS score. The proportion of individuals with cognitive impairment was significantly higher than that of those with normal cognition (*p* < 0.0001).

Table 1 summarizes the biochemical and cognition scores across the study groups. There was no significant difference in age across the groups (*p* = 0.441), with mean ages ranging from 66.5 to 69.2 years. The proportion of female participants was highest in the sufficient (69.81%) and insufficient (67.21%) groups compared to the deficient group (51.61%). Fasting insulin levels showed a statistically significant difference across groups (*p* = 0.014), with the highest mean in the vitamin D-deficient group (9.30 ± 5.66 µU/mL). HOMA-IR, an index of insulin resistance was elevated in the deficient and insufficient groups but did not reach statistical significance (*p* = 0.081). No significant differences were observed in fasting blood glucose, HbA1c, lipid profile or hemoglobin levels across groups. The 3MS cognitive scores were not significantly different among the groups (*p* = 0.308). Interestingly, the deficient group had the highest mean score (63.29 ± 26.11), whereas the sufficient group had the lowest (55.79 ± 25.76), although this was not statistically significant.

### 3.2. Association Between Vitamin D Status and Cognitive Impairment

When stratified by vitamin D status, the prevalence of cognitive impairment was highest among those with insufficient and sufficient vitamin D levels: the lowest prevalence of cognitive impairment was observed in the vitamin D-deficient group (61.29%), while it was similar and higher among the insufficient (77.04%) and sufficient (77.36%) groups (Table 2). There was no statistically significant association between vitamin D status and cognitive impairment in the unadjusted chi-square test (chi-square value = 3.96, *p*-value ≈ 0.138). Odds ratio (OR) analysis, using the vitamin D-deficient group as the reference, and odds of cognitive impairment were calculated: participants with vitamin D insufficiency or sufficiency had approximately twice the odds of cognitive impairment compared to those who were deficient, but the differences were not statistically significant (insufficient vs. deficient: OR—1.98, 95% CI was 0.79–4.96; and sufficient vs. deficient: OR—2.09, 95% CI was 0.83–5.29).

Table 3 depicts the amyloid beta levels and oxidative stress markers among the vitamin D status groups. SOD activity was significantly elevated in the vitamin D-deficient group (298.86 ± 283.60 U/mL) compared to the other groups (*p* = 0.0043,) probably due to compensatory antioxidant response in vitamin D deficiency. GPx activity also differed significantly (*p* = 0.045), with the highest levels seen in the vitamin D-insufficient group (86.51± 68.10 U/mL). TBARS, a marker of lipid peroxidation showed no significant difference among the groups (*p* = 0.500).

TBARS: thiobarbituric acid reactive substances; GPx: glutathione peroxidase; SOD: superoxide dismutase; Aβ: amyloid beta. *p* < 0.05 is considered statistically significant. Note: Due to non-normal distribution and high variability in SOD and GPx activity, *p*-values were calculated using the Kruskal–Wallis test.

Serum Aβ1-40, Aβ1-42 and the Aβ1-42/Aβ1-40 ratio did not show statistically significant differences across vitamin D categories. Although not significant, a gradual increase in Aβ1-42 levels was observed with declining vitamin D levels.

A correlation analysis test was used to investigate the association amongst age, vitamin D levels, cognitive ability (3MS), serum amyloid levels and oxidative stress markers. Table 4 shows that vitamin D had a weak and non-significant correlation with age, 3MS, amyloid markers and oxidative markers (GPx, TBARS). However, a statistically significant negative correlation was seen between vitamin D and SOD levels (r = −0.171, *p* = 0.039). This observation indicates that increased vitamin D levels were associated with decreased SOD activity, indicating a potential inverse relationship between vitamin D status and antioxidant enzyme activity.

Correlation analysis showed that 3MS scores were negatively associated with age (r = −0.2607, *p* = 0.0015), Aβ 1-40 (r = −0.4016, *p* = 0.0001), and Aβ 1-42 (r = −0.3025, *p* = 0.0002), and positively correlated with Aβ 1-42/Aβ 1-40 ratio (r = 0.2176, *p* = 0.0086) as shown in Table 5. 

### 3.3. Multivariate Logistic Regression Analysis

A multivariate logistic regression model was employed to identify independent predictors of cognitive impairment among the study participants. The model included demographic variables (age, sex), vitamin D, glycemic markers (HbA1c, HOMA-IR) and oxidative stress markers (SOD, GPx, TBARS).

Age factor was a significant predictor, with each year of age associated with 7% higher odds of cognitive impairment (adjusted OR = 1.07; 95% CI: 1.02–1.12; *p* = 0.004) as shown in Table 6. HbA1c also showed a similar trend, where a stronger association was observed between poor glycemic control and cognitive decline (OR = 1.58; 95% CI: 1.15–2.18; *p* = 0.006). Among the oxidative stress markers, SOD activity demonstrated a crucial neuro-protective role. Higher SOD activity was associated with lower odds of cognitive impairment (adjusted OR = 0.81; 95% CI: 0.68–0.96; *p* = 0.016). Vitamin D status showed a trend toward higher odds of normal cognition among both insufficient (OR = 2.02) and sufficient (OR = 2.11) groups when compared to the deficient group, though the associations did not reach statistical significance (*p* > 0.05). Sex (female vs. male), HOMA-IR, GPx activity and TBARS were not significantly associated with cognitive status in the adjusted model.

To assess the discriminatory ability of the multivariate logistic regression model in predicting cognitive impairment, an ROC curve was generated using age, HbA1c and SOD activity as predictors. The statistical analysis showed that the area under the ROC curve (AUC) was observed to be 0.85, thus indicating a fair predictive performance of the model.

### 3.4. Sensitivity Analysis: Amyloid Beta Biomarkers Stratified by Glycemic Status

Given the elevated overall glycemic burden in our cohort, we performed a sensitivity analysis to assess if clinical diabetic status moderated circulating amyloid profiles. Participants were stratified into non-diabetic (HbA1c < 6.5%, n = 95) and diabetic (HbA1c > 6.5%, n = 50) groups.

As detailed in Table 7, serum Aβ 1-40 and Aβ 1-42 levels were significantly elevated in the diabetic group compared to the non-diabetic group (Mann–Whitney *p* < 0.001). Crucially, the diabetic group exhibited a significantly reduced Aβ 1-42/Aβ 1-40 ratio (0.060 ± 0.02 vs. 0.074 ± 0.03; Mann–Whitney *p* = 0.04), indicating an altered peripheral amyloid profile in the presence of chronic glycemic dysregulation.

## 4. Discussion

This cross-sectional study investigated the complex associations between serum vitamin D status, cognitive performance, peripheral amyloid beta biomarkers and systemic oxidative stress markers among older adults attending a tertiary care hospital from a suburban population in South India. The primary finding of this study is that the vitamin D levels were not independently associated with cognitive performance as assessed by the Modified Mini Mental State Examination (3MS) in this cohort. Contrary to original hypothesis and several prominent observational reports in the literature that suggests that vitamin D deficiency influences cognitive performance, our data did not support this direct linear relationship between vitamin D status and cognitive function. Instead, the present study demonstrated a weak, non-significant association between serum vitamin D and 3MS cognitive scores (r = −0.0561, *p* = 0.5029). Further stratification of cognitive performance across the three vitamin D groups showed a paradoxical trend: interestingly, the vitamin D-deficient group exhibited the highest 3MS score (63.29 ± 26.11) and the lowest prevalence was observed in vitamin D-sufficient group (55.79 ± 25.76). This lack of direct association demonstrates that Vitamin D status does not serve as an independent predictor of global cognition in this clinically burdened sample. While many observational studies highlight a strong inverse association between vitamin D and cognitive decline, RCTs examining supplementation have produced mixed results, with some reporting mild cognitive benefits and others showing no effect [34,35]. Our study findings are in line with previous studies, which have reported inconsistent associations between vitamin D status and cognitive function [36,37,38]. While the literature outlines a multifaceted direct neuroprotective role for vitamin D that includes VDR-mediated synaptic plasticity, neurotrophic support and amyloid clearance pathways, these dynamic causal mechanisms cannot be demonstrated in our cross-sectional design and may have been masked by unmeasured confounding factors (such as APOE ε4 genotype, educational status, physical activity, dietary intake, socioeconomic status, sunlight exposure, seasonal variations and concurrent medication use) and the selection biases inherent to our combined clinical and suburban community sample.

Although vitamin D showed no statistical significance with cognitive impairment, it did exhibit a statistically significant correlation with circulating amyloid beta markers, Aβ 1-40 (r = −0.4016, *p* = 0.0001) and Aβ 1-42 (r = −0.3025, *p* = 0.0002) and was positively associated with Aβ 1-42/Aβ 1-40 ratio (r = −0.2176, *p* = 0.0086). These findings are consistent with the studies showing the relationship between amyloid markers and cognitive performance [39,40,41].

Beyond the amyloid burden, the chronic glycemic dysregulation also represented as an independent determinant of cognitive ability in older adults. In our multivariable logistic regression model, HbA1c was a highly significant predictor of cognitive impairment (adjusted OR = 1.58;95% CI: 1.15–2.18; *p* = 0.006) along with advanced age (adjusted OR = 1.07; 95% CI: 1.02–1.12; *p* = 0.004). This exceptionally high mean value of 6.96 across the entire study population, ranging from 6.92 in the insufficient group to 7.16 in the deficient group, indicates a substantial burden of chronic glycemic dysregulation in this cohort. Persistent hyperglycemia and underlying insulin resistance (evidenced by elevated fasting insulin of 9.30 ± 5.66 IU/mL and HOMA-IR of 2.58 ± 2.41 in the vitamin D-deficient group) are established mediators of neurodegenerative pathology. These observations underscore that metabolic and glycemic management may play a far more substantial role in preserving late-life cognitive function than single-nutrient approaches such as vitamin D supplementation. Recent clinical evidence has demonstrated an inverse association between HbA1c and cognitive performance [42,43,44,45]. Furthermore, longitudinal evidence in older adults with diabetes indicates that greater time spent within individualized HbA1c target ranges is associated with a lower risk of Alzheimer’s disease and related dementias.

In contrary, oxidative stress markers have shown diverse trends. Superoxide dismutase (SOD) activity was significantly elevated in the vitamin D-deficient group compared to the insufficient and sufficient groups (*p* = 0.0043). Similarly, glutathione peroxidase (GPx) was significantly elevated in cognitively impaired subjects within the deficient group (95.93 ± 51.90 U/mL, *p* = 0.038). The standard deviation for SOD in the deficient group is extremely large, nearly exceeding the mean, which indicates a highly skewed, non-normal distribution or considerable assay variability in competitive ELISA/enzymatic assays at these low ranges. Rather than representing a direct protective, compensatory response, elevated antioxidant enzyme activity may simply reflect upregulation secondary to chronic, systemic low-grade inflammation or a massive baseline oxidative stress burden. Without direct localized measurements of reactive oxygen species (ROS) or longitudinal follow-up, a definitive protective benefit cannot be inferred. Furthermore, TBARS demonstrated absolutely no significant differences across the vitamin D groups (*p* = 0.348) or cognitive groups (adjusted OR = 0.88, *p* = 0.083 in the multivariable model). Although TBARS is a primary endpoint for lipid peroxidation, circulating peripheral lipid peroxides are general systemic markers and may be relatively insensitive to localized, tissue-specific oxidative damage in the central nervous system. This finding of non-association of TBARS with vitamin D has been reported in previous studies [46,47,48]. The absence of a significant association between vitamin D status and TBARS levels may partly reflect the high baseline metabolic burden of our cohort, as indicated by a mean HbA1c of approximately 6.97%. The resulting high background oxidative stress may have limited the ability to detect subtle vitamin D-specific differences in systemic lipid peroxidation, thereby contributing to the observed null association.

The multivariate logistic regression analysis showcased age, higher HbA1c, and a lower SOD activity as independent predictors of cognitive impairment. Aging is a well-established risk factor for onset of dementia [49], and hyperglycemia also contributes to amyloid plaque deposition, cellular oxidative stress and thus neuronal dysfunction [50]. Insulin resistance has been shown to impair the neuronal glucose metabolism and may also potentiate amyloid aggregation and tau processing [51]. These observations are consistent with earlier studies connecting metabolic dysregulation to cognitive decline [52,53].

While this study found no direct association between serum vitamin D levels and global cognitive scores, its biochemical role in regulating amyloid pathology and oxidative stress suggests it may still serve as a modifiable risk factor for cognitive decline in older populations.

Our sensitivity analysis stratifying amyloid biomarkers by glycemic status provides compelling clinical evidence of metabolic–amyloid interactions. The significant elevation of both circulating Aβ 1-40 and Aβ 1-42 in diabetic participants, combined with a significantly reduced Aβ 1-42/Aβ 1-40 ratio, suggests that chronic hyperglycemia and insulin resistance may severely disrupt peripheral amyloid homeostasis. Elevated circulating glucose and insulin resistance may contribute to impaired amyloid-β clearance through metabolic pathways involving the insulin-degrading enzyme (IDE), which participates in the degradation of both insulin and amyloid-β. Such metabolic disturbances could potentially favor amyloid-β retention and accumulation. In this context, our findings highlight the potential importance of addressing overall metabolic health and glycemic control as modifiable factors for reducing amyloid burden and supporting cognitive health. This may have greater clinical relevance than focusing on isolated nutritional interventions, such as vitamin D supplementation, particularly in individuals with substantial metabolic risk.

Ultimately, longitudinal and interventional clinical trials are essential to establish causality and define the optimal vitamin D thresholds required for neuroprotection in late life.

### 4.1. Clinical Implementation

Cognitive decline and dementias like Alzheimer’s disease pathology have been linked to numerous factors, including glycemic regulation and oxidative stress, which are considered to be the modifiable risk factors. Focusing on these through appropriate interventions can be more advantageous than solely depending on vitamin D therapy for preserving cognitive health. Approaches that prioritize antioxidant support, lifestyle modifications, and metabolic re-arrangements may provide a more comprehensive strategy for sustaining healthy cognitive function in older adults.

### 4.2. Limitations

The cross-sectional design of our study inherently limits our ability to draw causal conclusions regarding the relationships between vitamin D, metabolic parameters, amyloid biomarkers, and cognitive function. Several critical confounding pathways could not be controlled in our models. First, we lacked genetic profiling for the apolipoprotein E (APOE) ε4 allele, which is the most potent genetic risk factor for sporadic Alzheimer’s disease and is independently associated with both peripheral Aβ kinetics and cognitive decline. Without APOE ε4 stratification, the observed associations between amyloid markers and 3MS scores cannot be fully disentangled from genetic predisposition. Second, we did not have exhaustive details on participants’ medication histories, specifically the use of statins, antihypertensive agents, or pre-existing over-the-counter vitamin D and calcium supplements, which can profoundly affect lipid metabolism, glycemic control, peripheral Aβ profiles and baseline cognitive status. Third, key environmental and behavioral determinants of vitamin D, including objective measurements of sunlight exposure, seasonal variation, physical activity, and precise dietary intake, were not captured. These omissions represent fundamental confounding issues that limit our ability to attribute cognitive or metabolic differences solely to serum 25(OH)D levels. Also, as this study did not include domain-specific neuropsychological testing, we could not assess the relationship between the studied biomarkers and specific cognitive domains, and we recommend comprehensive neuropsychological assessment in future studies. Lastly, our sample size of 145 individuals from a single tertiary center restricts the generalizability of our findings to the larger population.

## 5. Conclusions

In conclusion, the study showed no association between serum vitamin D status and cognitive performance in this cohort. Instead, cognitive impairment was strongly associated with elevated plasma Aβ 1-40 levels, reduced Aβ 1-42/Aβ 1-40 ratios, advanced age and poor glycemic control (HbA1c). These results highlight the robustness of peripheral amyloid biomarkers and metabolic control as key biological correlates of cognitive health. Furthermore, oxidative stress markers did not show significant associations with cognitive function, with the exception of GPx in individuals deficient in vitamin D. Future longitudinal and interventional studies are required to determine whether vitamin D influences these biological pathways and to evaluate the therapeutic efficacy of multi-domain metabolic interventions in preserving cognitive function in older adults.

## Figures and Tables

**Table 1:** Demographic, biochemical parameters, and 3MS score across the study groups.

Parameters	Vitamin D Sufficient (n = 53)		Vitamin D Insufficient (n = 61)		Vitamin D Deficient (n = 31)		*p*-Value
	Mean	SD	Mean	SD	Mean	SD	
**Sex (F − n) (%)**	37/69.81		41/67.21		16/51.61		
**Age (years)**	68.19	8.93	69.15	9.68	66.55	10.99	0.441
**FBS** **(mg/dL)**	97.98	47.92	103.05	47.91	109.54	77.22	0.688
**HbA1c**	6.97	1.47	6.92	2.07	7.16	2.25	0.806
**Fasting Insulin** **(µIU/mL)**	6.30	3.32	8.90	0.64	9.30	5.66	0.014 *
**HOMA-IR**	1.54	1.00	2.29	1.91	2.58	2.41	0.081
**Hemoglobin** **gm%**	12.77	1.77	12.82	1.73	13.03	1.74	0.970
**Total cholesterol** **(mg/dL)**	171.26	39.80	182.20	35.66	176.00	35.37	0.293
**HDL** **(mg/dL)**	42.87	9.10	43.98	6.64	43.42	9.04	0.766
**LDL** **(mg/dL)**	101.01	30.28	107.93	23.05	103.21	25.10	0.369
**VLDL** **(mg/dL)**	26.65	11.34	28.52	13.91	29.44	13.59	0.737
**Triglycerides** **(mg/dL)**	133.98	56.58	143.18	69.41	148.55	67.75	0.721
**Vitamin D** **(ng/mL)**	37.64	6.88	25.18	2.77	15.06	4.29	<0.0001 *
**3MS score**	55.79	25.76	59.47	23.97	63.29	26.11	0.308

FBS: fasting blood sugar; HbA1c: glycated hemoglobin; HOMA-IR: homeostatic model of assessment of insulin resistance; HDL: high-density lipoprotein; LDL: low-density lipoprotein; VLDL: very low-density lipoprotein; 3MS: Modified Mini-Mental State Examination. *p* < 0.05 is considered statistically significant. * Statistically significant.

**Table 2:** Prevalence of cognitive impairment in the study groups.

Vitamin D Status	Cognition Impairment (n, %)	Cognition Normal (n, %)
Vitamin D Deficient (n = 31)	19 (61.29%)	12 (38.71%)
Vitamin D Insufficient (n = 61)	47 (77.04%)	14 (22.96%)
Vitamin D Sufficient (n = 53)	41 (77.36%)	12 (22.64%)

**Table 3:** Amyloid beta levels and oxidative stress markers among the study groups.

Parameters	Vitamin D Sufficient (n = 53)		Vitamin D Insufficient (n = 61)		Vitamin D Deficient (n = 31)		*p*-Value
	Mean	SD	Mean	SD	Mean	SD	
**TBARS**	69.91	7.85	69.49	7.96	71.95	7.41	0.500
**GPx**	58.62	44.47	86.51	68.10	75.93	51.90	0.045 *
**SOD**	131.18	213.98	82.09	217.90	298.86	283.60	0.0043 *
**Aβ 1-40**	146.19	83.61	154.84	89.78	150.59	90.45	0.830
**Aβ 1-42**	8.35	4.63	9.33	4.38	10.19	7.54	0.214
**Aβ 1-42/Aβ 1-40**	0.064	0.026	0.071	0.035	0.075	0.040	0.631

* Statistically significant.

**Table 4:** Vitamin D correlation with age, 3MS Score, amyloid levels and oxidative stress markers.

Variable	Correlation “r”	*p*-Value
Age	0.0479	0.5674
3MS Score	−0.0561	0.5029
Aβ 1-40 (pg/mL)	0.0239	0.7751
Aβ 1-42 (pg/mL)	−0.0611	0.4651
Aβ 1-42/Aβ 1-40	−0.1344	0.1070
TBARS	−0.0905	0.2793
GPx	−0.0981	0.2402
SOD	−0.1714	0.0393 *

3MS: Modified Mini-Mental State Examination; Aβ: amyloid beta; TBARS: thiobarbituric acid reactive substances; GPx: glutathione peroxidase; SOD: superoxide dismutase. *p* < 0.05 is considered statistically significant. * Statistically significant.

**Table 5:** 3MS correlation with age, amyloid levels and oxidative stress markers.

Variable	Correlation “r”	*p*-Value
Age	−0.2607	0.0015
Aβ 1-40 (pg/mL)	−0.4016	0.0001
Aβ 1-42 (pg/mL)	−0.3025	0.0002
Aβ 1-42/Aβ 1-40	0.2176	0.0086

Aβ: amyloid beta; *p* < 0.05 is considered statistically significant.

**Table 6:** Multivariable logistic regression analysis for predictors of cognitive impairment in older adults.

Predictor	Adjusted OR	95% CI	*p*-Value
Age (per year increase)	1.07	1.02–1.12	0.004 **
Female (vs. male)	1.45	0.65–3.25	0.368
Vitamin D insufficient	2.02	0.71–5.72	0.182
Vitamin D sufficient	2.11	0.74–6.03	0.165
HbA1c (%)	1.58	1.15–2.18	0.006 **
HOMA-IR	1.22	0.93–1.62	0.142
SOD activity	0.81	0.68–0.96	0.016 **
GPx activity	0.92	0.80–1.05	0.210
TBARS	0.88	0.76–1.02	0.083

(Nagelkerke R^2^ = 0.46, model *p*-value (omnibus test) ≤ 0.001, ** Statistically highly significant. Hosmer–Lemeshow goodness-of-fit = *p* = 0.622) HbA1c: glycated hemoglobin; HOMA-IR: homeostatic model of assessment of insulin resistance; SOD: superoxide dismutase; GPx: glutathione peroxidase; TBARS: thiobarbituric acid reactive substances. *p* < 0.05 is considered statistically significant.

**Table 7:** Serum amyloid beta biomarkers stratified by glycemic status (non-diabetic vs. diabetic).

Variable	Non-Diabetic (HbA1c < 6.5%) n = 95	Diabetic (HbA1c > 6.5%) n = 50	*p*-Value
Aβ 1-40 (pg/mL)	131.34 ± 87.35	190.01 ± 71.29	<0.001
Aβ 1-42 (pg/mL)	8.45 ± 9.65	10.57 ± 4.05	<0.001
Aβ 1-42/Aβ 1-40	0.074 ± 0.03	0.060 ± 0.02	0.04

Aβ: amyloid beta; *p* < 0.05 is considered statistically significant.

## Data Availability

The data generated and/or analyzed during the current study are not publicly available due to privacy and ethical restrictions but may be made available from the corresponding author upon reasonable request, subject to applicable ethical and institutional requirements.
